# Abstract deliberation by visuomotor neurons in prefrontal cortex

**DOI:** 10.1038/s41593-024-01635-1

**Published:** 2024-04-29

**Authors:** Julie A. Charlton, Robbe L. T. Goris

**Affiliations:** 1 Center for Perceptual Systems, The University of Texas at Austin, Austin, TX USA; 2 Present Address: Princeton Neuroscience Institute, Princeton University, Princeton, NJ USA

**Keywords:** Decision, Neural encoding, Pattern vision

## Abstract

During visually guided behavior, the prefrontal cortex plays a pivotal role in mapping sensory inputs onto appropriate motor plans. When the sensory input is ambiguous, this involves deliberation. It is not known whether the deliberation is implemented as a competition between possible stimulus interpretations or between possible motor plans. Here we study neural population activity in the prefrontal cortex of macaque monkeys trained to flexibly report perceptual judgments of ambiguous visual stimuli. We find that the population activity initially represents the formation of a perceptual choice before transitioning into the representation of the motor plan. Stimulus strength and prior expectations both bear on the formation of the perceptual choice, but not on the formation of the action plan. These results suggest that prefrontal circuits involved in action selection are also used for the deliberation of abstract propositions divorced from a specific motor plan, thus providing a crucial mechanism for abstract reasoning.

## Abstract (web summary)

Charlton and Goris developed a new perceptual decision-making task for macaque monkeys and found that prefrontal circuits involved in action selection are also used for the deliberation of abstract propositions divorced from a specific motor plan.

## Main

Actions are guided by perceptual interpretations of the environment. Within a given context, perceptual interpretations may be stereotypically linked to specific actions. For example, when a driver in congested traffic sees the car ahead slow down, she will lift her foot from the gas pedal. When she sees the car speed up, she will instead press the gas pedal more firmly. Perceptual estimates of changes in car speed are imperfect. Deciding how to act in traffic therefore requires deliberation, especially when the changes are subtle. Deliberation here refers to the computational process of weighing evidence in favor of different choice options. Under static contextual circumstances, brain regions involved in action selection appear to represent such deliberation processes as a competition among possible action plans^1–3^. In the example above, this would mean that the deliberation process is implemented as a competition between a set of motor neurons responsible for lifting the foot and a second set responsible for pressing it down. In principle, this ‘intentional’ strategy can be used for many types of decisions that culminate in action^4–6^. Alternatively, perceptually guided decision-making could involve competition between a set of neurons responsible for perceiving the car ahead as slowing down, and a second set responsible for perceiving it as speeding up. The outcome of this deliberation process would then inform the ensuing action. The key distinction is that this type of deliberation involves more abstract propositions that are not linked to movements per se^7,8^.

**Fig. 1:**
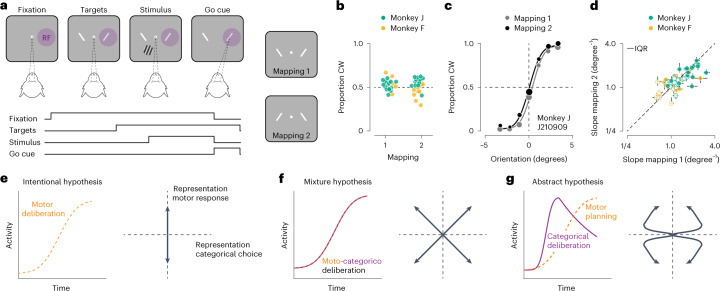
Flexible visual categorization: behavior and computational hypotheses. **a**, Orientation discrimination task, task sequence. After the observer fixates for 500 ms, two choice targets appear, followed by the stimulus. The observer judges whether the stimulus is rotated clockwise or counterclockwise relative to vertical and communicates this decision with a saccade towards the matching choice target. Correct decisions are followed by a juice reward. One of the choice targets is placed in the neurons’ presumed motor response field (RF) (Methods). The spatial organization of the choice targets varies randomly from trial to trial, giving rise to two stimulus-response mapping rules. **b**, Proportion of clockwise (CW) choices under both mapping rules for both animals. Each symbol summarizes the behavior from a single recording session. **c**, Psychophysical performance for monkey J in an example recording session. Proportion of CW choices for high-contrast stimuli is shown as a function of stimulus orientation under both mapping rules. Symbol size reflects the number of trials (total 3,582 trials). The curves are fits of a behavioral model (Methods). **d**, Comparison of orientation sensitivity (that is, the slope of the psychometric function, defined as the inverse of the s.d. of a cumulative Gaussian function) under both mapping rules for both monkeys (Methods). Each symbol summarizes data from a single recording session. Closed symbols, high-contrast stimuli; open symbols, low-contrast stimuli. Error bars reflect the interquartile range (IQR) of the estimate. **e**–**g**, Computational hypotheses (left; intentional hypothesis (**e**); mixture hypothesis (**f**); abstract hypothesis (**g**)) and associated neural representation motifs (right). There are four possible behavioral outcomes (that is, either a clockwise or counterclockwise choice, communicated with either a left or rightward saccade), resulting in four motifs per hypothesis.

Natural behavior occurs under many different contexts and therefore generally requires a flexible association between perceptual interpretation and motor response. It has been hypothesized that, when such flexibility is required, deliberation may consist of a competition among possible interpretations of the sensory environment rather than among possible action plans^9–12^. Here, we test this hypothesis using a task that requires flexible reporting of perceptual decisions. We trained two macaque monkeys (F and J) to judge whether a visual stimulus presented near the central visual field was oriented clockwise or counterclockwise from vertical (Fig. 1a–d). The monkeys communicated their judgment with a saccade to one of two peripheral visual targets. The meaning of each response option was signaled by the target’s orientation (clockwise versus counterclockwise), and was unrelated to its spatial position (one target was placed in the neurons’ estimated motor response field, the other on the opposite side of the fixation mark; Methods). Because the spatial configuration of the choice targets varied randomly from trial to trial, the task requires subjects to flexibly switch between two stimulus-response mapping rules (Fig. 1a). While the animals performed this task, we recorded extracellular responses from neural ensembles in the prearcuate gyrus (Supplementary Fig. 1), an area of prefrontal cortex (PFC) involved in the selection of saccadic eye movements^13,14^ that represents visuomotor deliberation^2,15^. Importantly, the choice targets in our task are presented before the onset of the stimulus, allowing the subjects to either adopt an intentional or an abstract deliberation strategy.

We found that the activity of many units was not only predictive of the upcoming motor response, but also of the categorical meaning of the perceptual choice. To gain further insight into the evolving decision state of the monkeys, we computed a time-varying decision variable (DV) composed of two dimensions that reflect the ‘categorical’ and ‘motor’ components of the neural population response. We demonstrate that, following stimulus onset, population activity initially represents the formation of a perceptual choice before transitioning into the stereotypical representation of the upcoming motor response. As predicted by theoretical models of decision-making, the formation of the perceptual choice reflected a graded representation of evidence, informed by both the current sensory input and stimulus expectations. This was not true of the evolving representation of the motor plan, which unfolded later, in an orthogonal dimension. Together, these results suggest that prefrontal circuits involved in action selection also support deliberation among abstract propositions.

## Results

### Behavior and single unit responses

**Extended Data Fig. 1:**
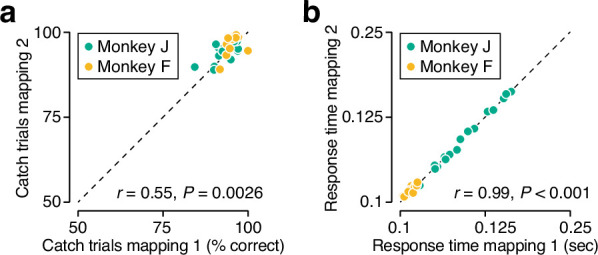
Further comparison of psychophysical performance under both mapping rules. (**a**) Proportion correct judgements for the easiest stimulus conditions (that is, the two most extreme stimulus orientations, a.k.a. ‘catch’ trials). Only high contrast trials were included in the analysis. Each symbol summarizes the behavior from a single recording session. Task performance consistently approached the level expected from a flawless observer without attentional lapses (that is, 100% correct) and did not differ across both mapping rules (median difference in task performance: 1.6%, *P* = 1, 2-sided Wilcoxon signed-rank test). The positive association across both mapping rules indicates that the fraction of guesses may vary across sessions, but is stable across mapping rules. (**b**) The average response time across all trials completed within a single recording session. Response time is measured relative to the go cue. *r* is Spearman correlation. P is the probability that the correlation differs from zero using a two-sided test.

Both monkeys learned to categorize stimulus orientation successfully under the two mapping rules. Their perceptual choices were distributed evenly among both response alternatives (Fig. 1b), and depended lawfully on stimulus orientation (Fig. 1c). They made few errors in the easiest stimulus conditions (monkey F = ±3.75 degrees, median performance, 96.25% correct; monkey J = ±3.3 degrees, median performance, 94.38% correct; Extended Data Fig. 1a). The spatial location of the choice targets varied across recording sessions, impacting the animals’ orientation sensitivity. It did so in similar fashion under both mapping rules (median difference in orientation sensitivity: monkey J = 4.4%, *P* = 0.45, *N* = 32; monkey F = 4.7%, *P* = 0.38, *N* = 26; Wilcoxon signed-rank test; Fig. 1d). This pattern was also evident in the animals’ saccade latency (Extended Data Fig. 1b). Together, these results suggest that, within each session, the quality and duration of the decision process did not vary meaningfully across the two mapping rules.

What is the nature of the decision process that underlies this flexible behavior? One viable strategy would be to evaluate which saccadic eye movement is more likely to be correct (the ‘intentional’ hypothesis; Supplementary Fig. 2). In principle, this strategy can be instantiated by oculomotor neural circuits. Alternatively, the deliberation may concern which categorical choice option is most likely to be correct (the ‘abstract’ hypothesis; Supplementary Fig. 2). However, it is not clear which neural circuits would instantiate this computation. Finally, the deliberation process might involve joint consideration of the stimulus category and the corresponding motor plan (the ‘mixture’ hypothesis; Supplementary Fig. 2). We designed the task such that each of these strategies produces a qualitatively distinct ‘motif’ of population activity that represents the unfolding visuomotor deliberation process. The motifs are defined by the joint evolution of activity related to the upcoming categorical choice and the upcoming saccade direction (Fig. 1e–g). We thus set out to characterize the dynamic structure of population activity in PFC while the animals generated this behavior.

**Fig. 2:**
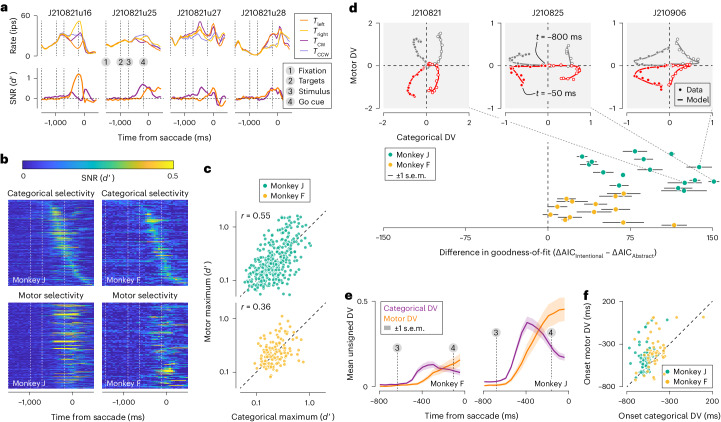
Dynamics of neural activity in PFC during flexible visual categorization. **a**, Temporal evolution of firing rate (top) and response selectivity (bottom) of four jointly recorded units (ensemble size, 29 units). Spikes were counted using 50-ms-wide counting windows and averaged across trials that either shared the same saccade direction (dark versus light orange) or the same perceptual category (dark versus light purple). Vertical lines indicate the average time of critical task events. ips, impulses per second; CCW, counterclockwise. **b**, Temporal evolution of response selectivity for the chosen perceptual category (top) and the saccade direction (bottom) of all units recorded from monkey J (left) and monkey F (right). In all displays, units are ranked according to the timing of their maximal categorical selectivity. Vertical lines indicate the average time of critical task events. **c**, Maximal response selectivity for saccade direction plotted against maximal selectivity for the categorical choice on logarithmic axes. *r*, Spearman correlation; *N* = 363 units for monkey J and 126 units for monkey F. **d**, Top: example DV trajectories during a 750-ms epoch preceding saccade initiation for three recording sessions. Symbols represent cross-validated data-based estimates and lines represent the fit of a model instantiating the abstract hypothesis (Methods). The gray and red lines correspond to left and right saccades and filled versus open dots correspond to clockwise versus counterclockwise decisions. Bottom: comparison of goodness-of-fit of two descriptive models instantiating the abstract and intentional hypothesis. Error bars were computed across each recording session’s four trajectories; *N* = 16 recording sessions for monkey J and 13 sessions for monkey F. **e**, Average observed unsigned DV trajectories. Each recording session contributes two unsigned trajectories to this plot. Vertical lines indicate the average time of critical task events. **f**, Onset of the motor DV plotted against onset of the categorical DV for all trajectories (Methods).

**Extended Data Fig. 2:**
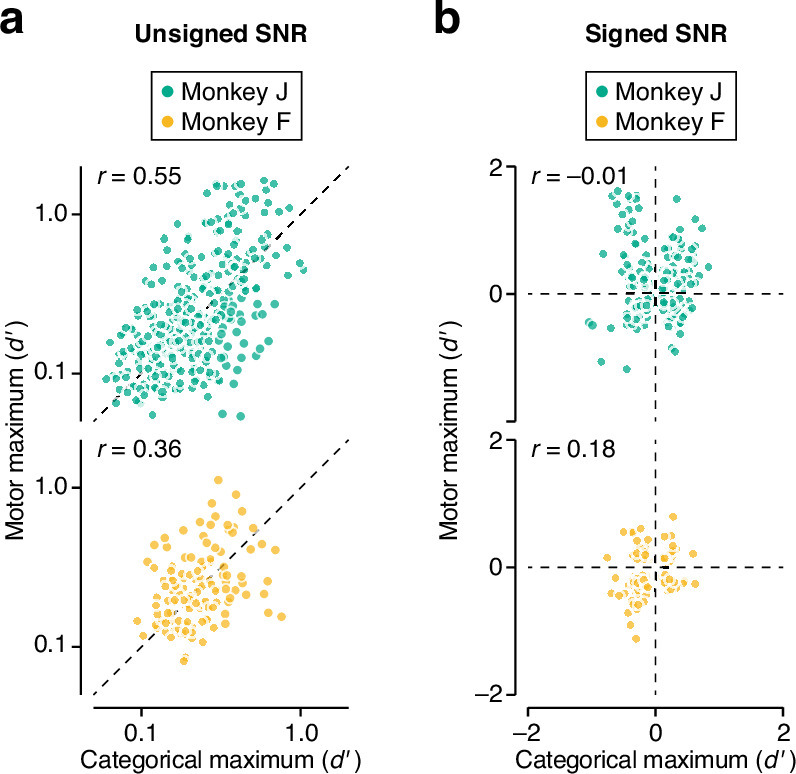
PFC neurons exhibit mixed selectivity for stimulus category and saccade direction. (**a**) Maximal unsigned response selectivity for saccade direction plotted against maximal selectivity for the categorical choice on logarithmic axes (same as Fig. 2c of the main paper). (**b**) Most extreme signed response selectivity for saccade direction plotted against maximal selectivity for the categorical choice on linear axes. For both monkeys, every quadrant in the plot is occupied.

Consider the activity of four simultaneously recorded units. We targeted neurons whose motor response field was likely to overlap with one of the choice target locations (Methods). Grouping trials by saccade direction confirmed that the activity of many units was predictive of the upcoming motor response (Fig. 2a, top (dark versus light orange)). Grouping the same trials instead by saccade meaning revealed that the activity of many units was also predictive of the categorical choice (Fig. 2a, top (dark versus light purple)). The temporal evolution of choice-related activity differed across units, complicating a functional interpretation (Fig. 2a, bottom). But note that, in most cases, categorical selectivity peaked before the go cue (monkey F, 83 of 126 units; monkey J, 243 of 363 units), while motor selectivity peaked after the go cue (monkey F, 79 of 126 units; monkey J, 239 of 363 units; Fig. 2b). This pattern suggests that these predictive signals may be separated in time. The same units tended to exhibit both types of choice selectivity. Specifically, the larger the peak motor selectivity was, the larger the peak categorical selectivity tended to be (Fig. 2c; Spearman rank correlation: monkey J = 0.55, *P* < 0.001; monkey F = 0.36, *P* < 0.001). However, there was no obvious relationship between the units’ preferred saccade direction and their preferred stimulus category (Extended Data Fig. 2). Such mixed selectivity is thought to offer substantial computational advantage over specialized responses for implementing flexible input–output mappings as required for our task^16–18^. Note that variability in preferred saccade direction implies variability in the alignment of the units’ response fields with the choice targets. This alignment is a factor that may impact response properties of visuomotor neurons^19^.

### Dynamic population representation motifs

**Extended Data Fig. 3:**
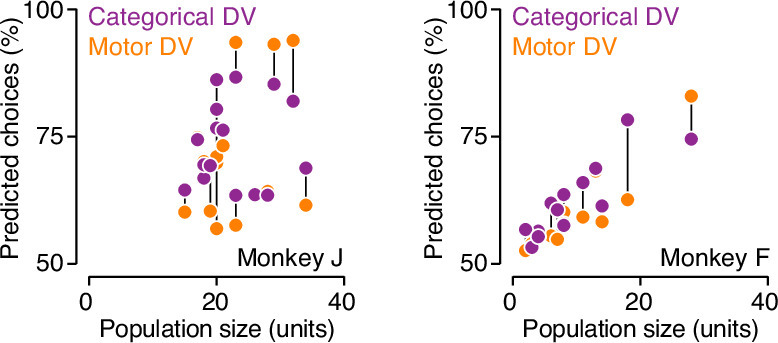
Performance of the categorical and motor DV against population size for both monkeys. (Left: monkey J, Right: monkey F). We used a 750 ms counting window (–800 to −50 ms relative to saccade onset), computed the optimal projection planes for each dataset, and calculated the fraction of trials for which the resulting DV accurately predicted choice outcome. Higher values indicate a stronger population representation of future choice outcome. For both monkeys, DV performance strongly varied across experiments. In general, population size was positively associated with DV performance (the more units we recorded simultaneously, the better the choice outcome could be predicted).

To obtain a perspective on neural population activity during flexible visual categorization, we decoded a time-varying DV from jointly recorded responses (Methods). We used a method that specifically extracts the choice-related component from high-dimensional neural activity. The decoded DV indicates how well the subject’s upcoming choice can be predicted from a 50 ms bin of neural ensemble activity^20^ (Extended Data Fig. 3). Each behavioral choice is summarized by two independent binary variables: the chosen saccade direction and the corresponding perceptual meaning. Likewise, the DV is composed of two independent dimensions. Its temporal structure defines the population representation motif and may thus disambiguate the nature of the decision process (Fig. 1e–g).

**Fig. 3:**
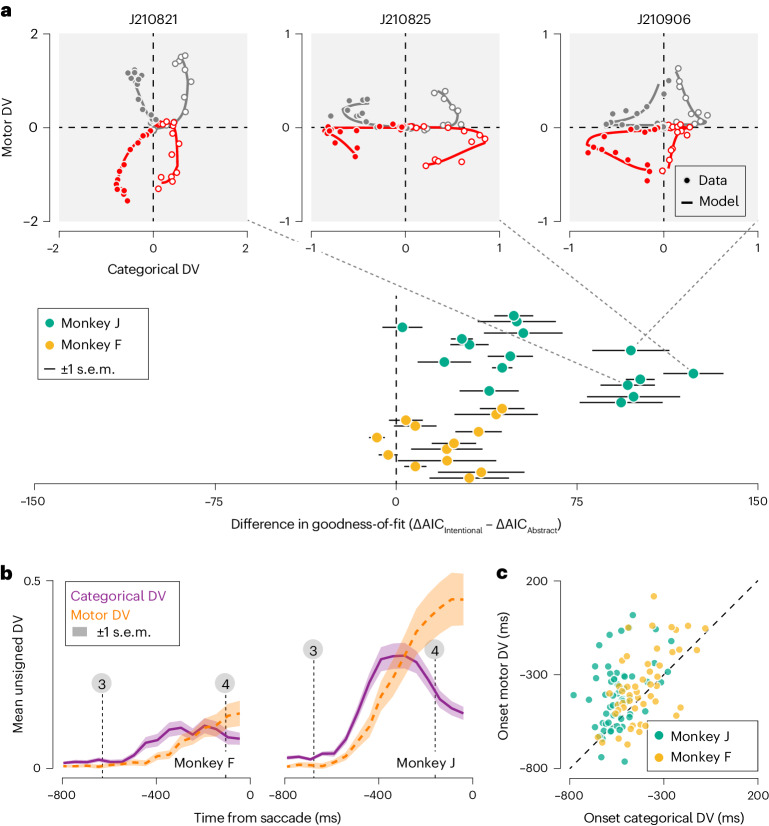
Dynamics of neural activity in PFC during deliberation of zero-signal stimuli. **a**, Top: example DV trajectories during a 750-ms epoch preceding saccade initiation for three recording sessions. Only trials that involved the zero-signal stimulus (stimulus orientation = 0 degree) were included in this analysis. Bottom: comparison of goodness-of-fit of two models instantiating the abstract and intentional hypothesis. Same plotting conventions as Fig. 2d. **b**, Average observed unsigned DV trajectories (zero-signal trials only). Same plotting conventions as Fig. 2e. **c**, Onset of the motor DV plotted against onset of the categorical DV for all trajectories (zero-signal trials only).

**Extended Data Fig. 4:**
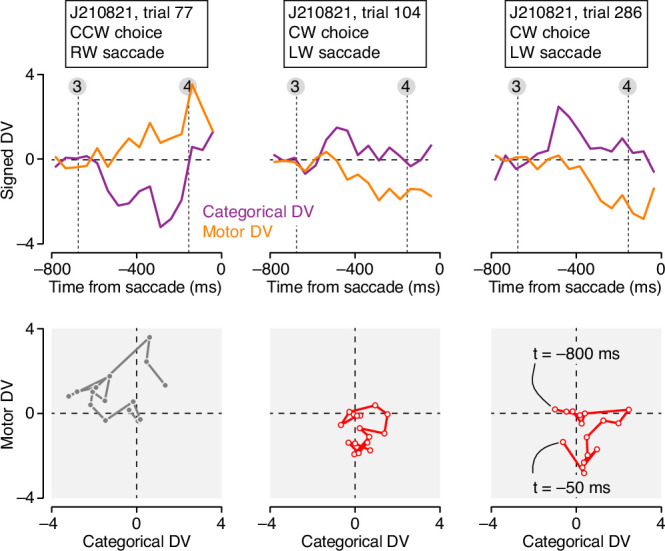
Example single trial DV trajectories during a 750 ms epoch preceding saccade initiation. These three trials all come from the same recording session. The top row shows the temporal evolution of the categorical and motor DV (purple vs orange). The bottom row shows the same data, plotted against each other. The leftmost trial resulted in a counterclockwise choice (shown in gray), communicated with a rightward saccade. The middle and rightmost example both resulted in a clockwise choice (shown in red), communicated with a leftward saccade. Same plotting conventions as Fig. 2d.

**Extended Data Fig. 5:**
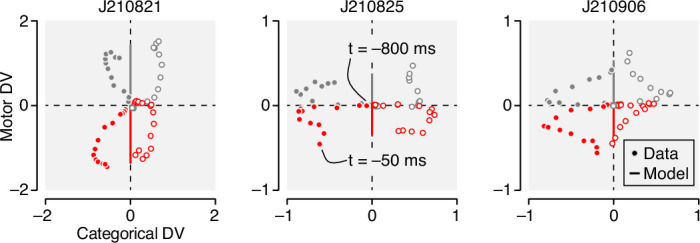
Example DV trajectories during a 750 ms epoch preceding saccade initiation for three recording sessions. Symbols represent cross-validated data-based estimates, solid vertical lines the fit of a model commensurate with an intentional strategy. This model cannot capture temporal structure in the categorical dimension and hence provides a poor fit to the data (compare with the fit of the abstract model to the same data, shown in Fig. 2d of the paper).

**Extended Data Fig. 6:**
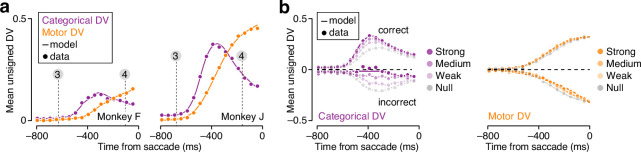
Comparison of model-predicted and observed DV trajectories. (**a**) Lines show the average unsigned DV trajectories predicted by an unconstrained model. Each recording session contributes two unsigned trajectories to this plot. Symbols show the average observed values (same data as plotted in Fig. 2e in the main paper). Vertical lines indicate the average time of critical task events. These model fits were used to estimate the onset time for each trajectory (shown in Figs. 2f and 3c). (**b**) Lines show the fit of a descriptive model to the average unsigned DV trajectories split by choice accuracy (top: correct trials; bottom: incorrect trials) and stimulus strength (that is, orientation). Symbols show the average observed values (same data as plotted in Fig. 4b in the main paper). These model fits were used to estimate the peak time of each trajectory (shown in Fig. 4f).

**Extended Data Fig. 7:**
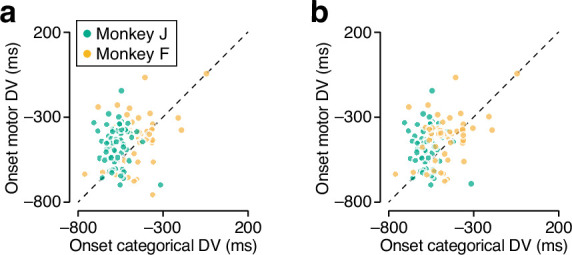
Alternative quantification of DV onset time. In the main paper, we define DV onset time in relative terms (that is, the time point by which the cumulative Gaussian trajectory exceeds its fifth percentile). This implies that the corresponding DV ‘threshold’ will depend on the DV’s dynamic range and will thus often differ for the categorical and motor dimension. Here, we considered an absolute alternative. For each trajectory, we imposed the same DV threshold on both dimensions (that is, 10% of the dynamic range of the dimension with the smallest range). As expected, the relative and absolute onset estimates differ somewhat. Both statistics support the conclusion that the categorical DV tends to have an earlier onset time than the motor DV. (**a**) Onset time for DV trajectories computed using all trials (as in Fig. 2f). (**b**) Onset time for DV trajectories computed using only zero-signal trials (as in Fig. 3c).

Trajectories were noisy at the single trial level (Extended Data Fig. 4), but exhibited clear structure when averaged across all trials that resulted in the same behavioral outcome. Consider the trial-averaged DV trajectories of three example ensembles. To a first approximation, an initial excursion along the categorical dimension is followed by an excursion in the motor dimension (Fig. 2d, top, symbols). Quantitatively, these trajectories are well captured by a model that describes an abstract decision strategy (Fig. 2d, top, curves). In contrast, a model consistent with an intentional decision strategy provides a poorer fit to the same data as it cannot capture temporal structure in the categorical dimension (Extended Data Fig. 5). This pattern held true for each recorded ensemble (Fig. 2d, bottom; Methods). A model consistent with the mixture hypothesis rivaled the abstract model for one monkey, but performed systematically worse for the other animal (monkey F, mixture model preferred over abstract model for 8 of 13 datasets; monkey J, 1 of 16; Methods). To further disambiguate between the abstract and mixture hypotheses, we studied the temporal relationship between the two DV dimensions. Key to the mixture hypothesis is the simultaneous evolution of decision-related activity in both dimensions (Fig. 1f). However, the categorical DV tended to precede the motor DV. This can be seen in the average unsigned observed DV trajectories, obtained by inverting the trajectories associated with ‘counterclockwise’ and ‘left’ choices and grouping these with the ‘clockwise’ and ‘right’ trajectories, respectively. In both monkeys, the average unsigned categorical DV begins rising within 150 ms following stimulus onset, well before the average unsigned motor DV begins to rise (Fig. 2e). To investigate whether this pattern was also evident at the level of individual DV trajectories, we fit a version of the abstract model that did not constrain the motor DV to follow the categorical DV (Methods). The resulting fits closely resembled the observed data (Extended Data Fig. 6a), allowing us to estimate the onset time of the rise of each DV rise in a systematic manner (Methods). In most individual model-predicted trajectories, the categorical DV appears to begin rising before the motor DV (monkey F, 33 of 52 trajectories; monkey J, 55 of 64 trajectories; Fig. 2f). Quantifying onset time in a different manner produced a similar outcome (Extended Data Fig. 7). Restricting these analyses of the DV trajectories to the fully ambiguous stimulus condition (stimulus orientation, 0 degrees) yielded comparable results, suggesting that these patterns of neural activity are intimately related to the unfolding decision process, rather than to underlying physical stimulus differences as such (Fig. 3). We conclude that the dynamics of the DV are incompatible with the intentional hypothesis and slightly favor the abstract model over the mixture model.

### Neural signatures of deliberation

We have shown that the temporal structure of population activity in PFC is incompatible with the hypothesis that intentional deliberation underlies the monkeys’ flexible behavior. It is also incompatible with a task-specific variant of this hypothesis (a spatial match-to-sample strategy; Supplementary Fig. 3), and offers only moderate support for the mixture hypothesis. Instead, our analysis favors the hypothesis that abstract deliberation underlies the monkeys’ flexible behavior. If this interpretation is correct, then the categorical DV ought to exhibit key signatures of deliberation. Moreover, these signatures should not be present in the motor DV. This prediction is unique to the abstract hypothesis (Fig. 1e–g), and thus offers a strong test of our proposed interpretation.

**Fig. 4:**
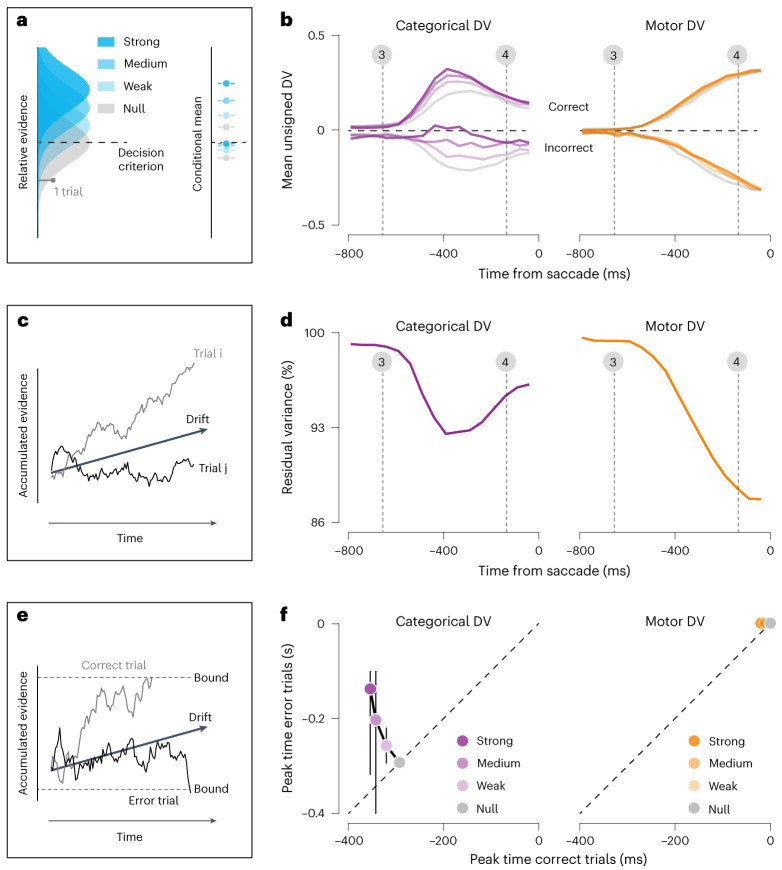
Effects of stimulus strength and choice accuracy on the DV. **a**, Binary decisions are often modeled as arising from a process whereby relative evidence (that is, the accumulated evidence that favors one response alternative over the other) is compared with a fixed criterion. Left: repeated presentations of the same condition elicit variable amounts of evidence, giving rise to an evidence distribution across many trials (four examples shown). Stronger stimuli result in a smaller overlap of the evidence distribution with the criterion, thus yielding more correct decisions. Right: average relative evidence split by choice accuracy (top, correct trials; bottom, incorrect trials) and stimulus strength. **b**, Average unsigned observed DV trajectories split by choice accuracy (top, correct trials; bottom, incorrect trials) and stimulus strength (that is, orientation; Methods). Data of all recording sessions were pooled. Vertical lines indicate the average time of critical task events as in Fig. 2a. **c**, If the accumulated evidence is obtained through temporal integration, cross-trial variance of the DV trajectory grows with integration time—a phenomenon known as ‘diffusion’. **d**, Variance of the residuals plotted against time. Data of all recording sessions were pooled (Methods). **e**, If the decision process is terminated by hitting a bound, average decision time depends on both choice accuracy and stimulus strength. **f**, Comparison of peak time of the average DV trajectories for incorrect and correct trials. Error bars, IQR of the estimate based on a 1,000-fold bootstrap (Methods).

The simplest theoretical models of decision-making hold that subjects solve binary decision-making tasks by comparing the evidence that favors one response alternative over the other with a fixed criterion^21^. Due to noise, repeated presentations of the same stimulus elicit different evidence estimates and may therefore result in different decision outcomes (Fig. 4a, left). When averaged across many trials, this deliberation process gives rise to a graded representation of relative evidence that varies with stimulus strength and differs for correct and incorrect decisions (Fig. 4a, right). For this reason, evidence estimates are thought to not only inform decision outcome, but also determine a subject’s commitment to an evolving decision^2,3^ and factor into their confidence in a decision^22,23^. If the neural populations we recorded from are involved in the deliberation process, their activity should thus reflect a graded representation of evidence. The issue at stake is whether this representation manifests in the motor DV, the categorical DV, or both.

**Extended Data Fig. 8:**
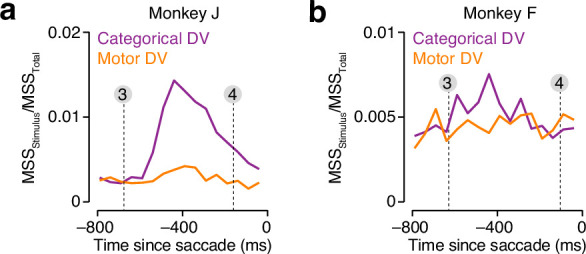
Fraction of DV variance explained by stimulus strength, after controlling for choice outcome (correct vs incorrect). (**a**) The average fraction of variance due to stimulus strength plotted against time for the categorical and motor DV (purple vs orange) for Monkey J. Vertical lines indicate the average time of critical task events. Following stimulus onset, variance in the categorical DV is increasingly explained by stimulus strength. This trend peaks around –400 ms, after which the curve returns to baseline. Variance in the motor DV does not reflect a prominent effect of stimulus strength. (**b**) Same analysis for monkey F. MSS mean sum of squares.

Consider the temporal evolution of the average unsigned DVs, split by stimulus strength and choice accuracy (Fig. 4b). Dividing trials across this many conditions dilutes the statistical power of the analysis. To compensate for this, we pooled data of both monkeys (Methods). As can be seen, approximately 150 ms after stimulus onset, the sign and amplitude of the categorical DV begin to match the theoretical prediction of evidence representation. Specifically, the categorical DV achieves more extreme values for correct decisions based on stronger stimuli but exhibits the opposite order for incorrect decisions (Fig. 4b, left). This pattern becomes increasingly prominent over the next 200 ms. In contrast, the amplitude of the motor DV does not appear to reflect the strength of the evidence supporting the choice that informed the upcoming saccade (Fig. 4b, right). Its stereotypical nature suggests that it represents a ‘pure’ motor plan. A statistical analysis of the temporal evolution of DV variance confirmed that, for both monkeys, stimulus strength had a larger impact on the categorical DV than on the motor DV (Extended Data Fig. 8).

Deliberation often takes time. Dynamical models of decision-making attribute this to a decision-making strategy in which momentary evidence is accumulated over time^24,25^. Due to noise, accumulated evidence exhibits ‘diffusion’, meaning that its cross-trial variance grows with integration time (Fig. 4c). A neural signature of this effect can sometimes be seen in the timecourse of residual response variance (that is, response variance that is not due to choice outcome or stimulus strength)^26^. Consider the residual variance of the categorical and motor DVs (Methods). In both cases, it is maximal early on in the trial and begins decreasing following stimulus onset (Fig. 4d). For the categorical DV, this initial decrease is followed by a rise that appears to last approximately 350 ms. This may be a signature of diffusion and, as such, suggests that animals integrated noisy stimulus orientation estimates over time^27^. The motor DV does not exhibit this effect. The more time progresses, the more stereotyped the representation of the upcoming motor action becomes (Fig. 4d).

If evidence is integrated over time, what terminates the deliberation process? One popular idea is that evidence is integrated until it reaches a bound. Under such a process, correct decisions are associated with shorter deliberation time than incorrect decisions (Fig. 4e)^24,25^. Consistent with this, the categorical DV trajectories appear to reach their most extreme value more quickly for correct than for incorrect decisions (Fig. 4b, left). The motor DV does not display this pattern (Fig. 4b, right). This visual impression was validated by a quantitative analysis (Fig. 4f, Extended Data Fig. 6b and Methods). This pattern suggests that the deliberation process typically continued until the categorical DV reached a bound. This event resulted in commitment to a perceptual choice, after which the categorical DV started to decay while the saccadic response used to communicate the decision was being prepared.

### Relationship between categorical and motor DV

**Fig. 5:**
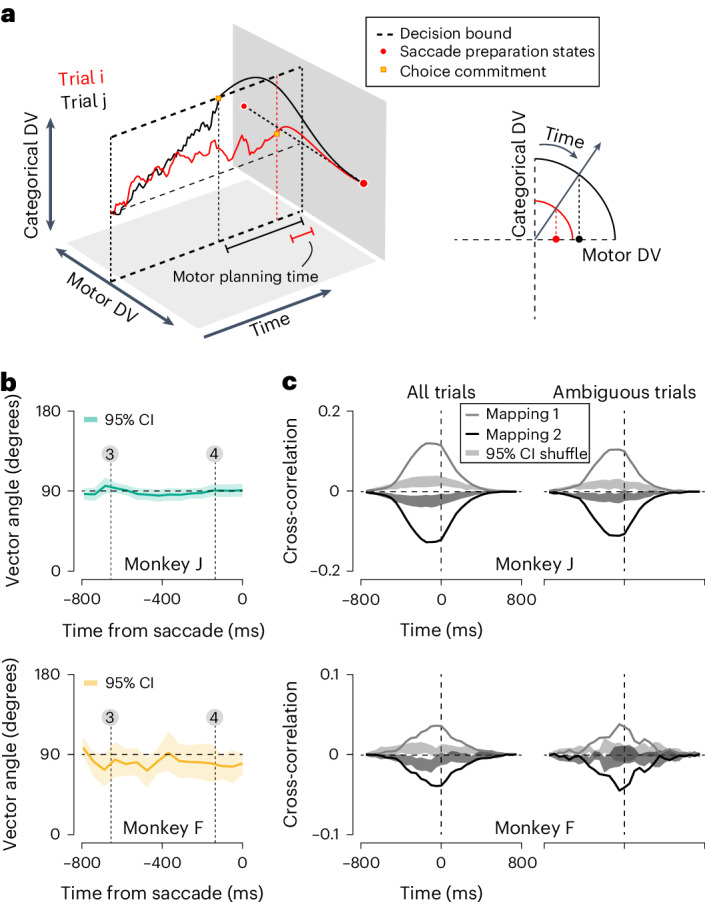
Relation between the categorical and motor DV dimensions. **a**, Illustration of an idealized bounded evidence integration process, followed by rotation towards a stereotyped saccade preparation state. Left: categorical and motor DV evolve in an orthogonal subspace. Trials that culminate in a higher value of the categorical DV will have a different starting point of the rotation onto the motor DV (black versus red curve). Right: if rotation happens at constant angular speed, categorical DV differences will be reflected in ensuing motor DV differences (black versus red line). **b**, Average vector angle between the categorical and motor DV projection planes as a function of time for both monkeys (top versus bottom). Band illustrates 95% CI. **c**, Cross-correlation between categorical and motor DV, split out by the mapping rule (black versus gray) for all trials (left), and the zero-signal trials (right). Data of both monkeys were analyzed separately (top versus bottom). Band illustrates 95% CI for a shuffled version of the data in which the categorical DV of each trial was paired with the motor DV of the next trial.

How are the categorical and motor DV related? We hypothesized the following. Deliberation unfolds in an abstract categorical dimension and resembles a bounded evidence accumulation process. When a bound is reached, or when time is up, the accumulated evidence is mapped onto a stereotypical saccade preparation state. This requires mapping the representation along the categorical dimension onto an orthogonal motor dimension (Fig. 5a, left). One neurally plausible way of doing this is through rotation^28,29^. The mapping process might give rise to a systematic relationship between the categorical and motor DV. For example, if it is instantiated as rotation at constant angular speed, then the motor DV will echo the strength of the categorical DV at the time of choice commitment (Fig. 5a, right).

**Extended Data Fig. 9:**
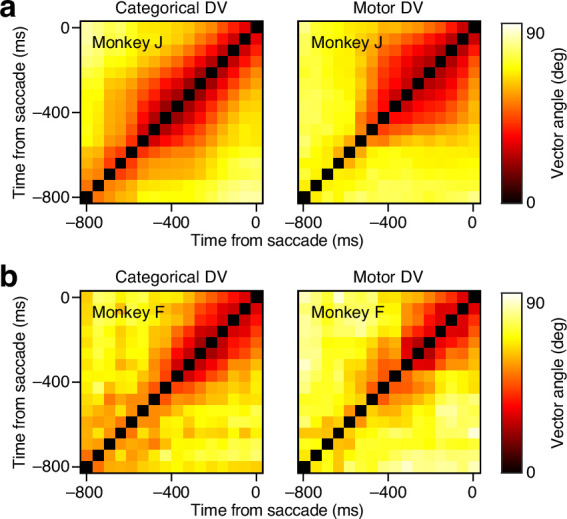
Temporal evolution of the categorical and motor projection planes. (**a**) Average vector angle between the projection planes computed at different time points for the categorical and motor DV (left vs right) for monkey J. The dark band around the line of unity indicates that the orientation of the categorical projection plane is fairly stable during the time of stimulus deliberation. This same pattern is evident in the motor DV, though shifted to a later point in time. It indicates a stable orientation of the motor projection plane during the time of saccade planning and preparation. (**b**) Same analysis for monkey F.

To test the orthogonality of the categorical and motor dimensions, we first computed the vector angle between the associated projection planes (Methods). Throughout the epoch of interest, the vector angle closely approximated 90 degrees for both monkeys (Fig. 5b). This pattern was intended by the task’s design, but was not guaranteed to emerge as it requires sufficiently unbiased choice behavior. We also studied the temporal evolution of the projection planes’ orientation and found them to be fairly stable through time (Extended Data Fig. 9). These analyses confirm that, during the decision-making process, PFC population activity contained orthogonal and stable representations of the upcoming perceptual choice and motor response.

To quantify the relationship between the categorical and motor DV, we next computed their cross-correlation function on a trial-by-trial basis and averaged the resulting functions across all trials that shared the same mapping rule (Methods). For both monkeys, this revealed an association between the categorical and motor DV beyond chance expectation (Fig. 5c). The cross-correlation function has several noteworthy features. First, its sign depends on the mapping rule. This makes sense given that the same perceptual choice needs to be reported with opposite saccade directions under the two mapping rules. Second, the cross-correlation function was asymmetric with respect to the time reversal operation (that is, the change of the sign of the time lag). It peaked at a time lag of roughly −100 ms and did not differ from chance before −400 ms or after 200 ms (Fig. 5c, left). This asymmetry hints at the presence of a causal relationship as it means that past values of the categorical DV predict present or future values of the motor DV. Restricting this analysis to the fully ambiguous stimulus condition yielded nearly identical results, suggesting that this pattern is an inherent property of the unfolding decision-making process (Fig. 5c, right). In sum, the relation between the categorical and motor DV is strong enough to manifest as a nonzero time-shifted correlation (Fig. 5c), but not so strong as to give rise to a graded representation of evidence in the motor DV (Fig. 4b).

### Impact of statistical regularities in the environment

Perceptual decisions are determined not only by the present sensory input. They are also shaped by expectations that reflect previously experienced statistical regularities in the environment^30,31^. Knowledge of such regularities (‘prior knowledge’) provides evidence that bears on challenging visual categorization problems. In theory, it can therefore be leveraged to improve the quality of uncertain decisions. Ample empirical evidence demonstrates that humans and other animals heavily exploit prior knowledge for perception^31,32^, action^33,34^ and cognition^35,36^.

**Fig. 6:**
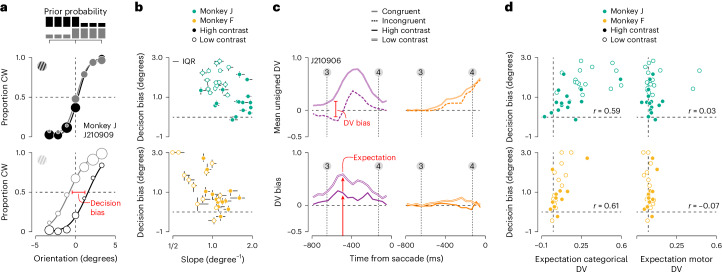
Effects of prior stimulus expectation on the DV. **a**, Psychophysical performance for monkey J in an example recording session. Proportion of clockwise choices is plotted as a function of stimulus orientation under both stimulus priors (black versus gray), split by stimulus contrast (top, high-contrast trials; bottom, low-contrast trials). Symbol size reflects the number of trials (total of 1,707 high-contrast trials and 1,875 low-contrast trials). The curves are fits of a behavioral model (Methods). **b**, Decision bias plotted as a function of orientation sensitivity, defined as the inverse of the s.d. of a cumulative Gaussian function, for both monkeys (top, monkey J; bottom, monkey F). Each symbol summarizes data from a single recording session. Closed symbols, high-contrast stimuli; open symbols, low-contrast stimuli. Error bars reflect the IQR of the estimate. **c**, Top: average unsigned DV trajectories split by choice congruency for an example recording session. Only low-contrast trials are included. Bottom: DV bias in the example dataset for high- and low-contrast trials. Categorical DV is shown on the left and motor DV is shown on the right. **d**, Decision bias plotted as a function of stimulus expectation for both monkeys. Same plotting conventions as in **b**.

We wondered how prior knowledge impacts PFC population representations during flexible visual categorization. To investigate this, we designed the task such that blocks of trials in which clockwise stimuli were over-represented alternated with blocks in which counterclockwise stimuli were over-represented (Methods). We additionally varied stimulus contrast. The current latent state of each trial was cued to the monkey through the shape of the fixation mark (Methods). When the stimulus contrast was high, perceptual orientation estimates were more certain, and the impact of the prior on the choice behavior was often small (Fig. 6a, top). When the stimulus contrast was low, perceptual orientation estimates were less certain, as evidenced by the shallowing of the psychometric function (Fig. 6a, bottom; median reduction in orientation sensitivity: monkey J = 46.4%, *P* < 0.001; monkey F = 40.7%, *P* < 0.001; Wilcoxon signed-rank test). As a consequence, the impact of the prior on the decision grew, giving rise to increased separation between the prior-specific psychometric functions, hereafter termed ‘decision bias’ (Fig. 6a, top versus bottom; median increase in decision bias: monkey J = 63.3%, *P* = 0.0013; monkey F = 68.6%, *P* = 0.04). In general, both monkeys tended to make more biased decisions under task conditions associated with lower orientation sensitivity (Fig. 6b; Spearman rank correlation: monkey J = −0.42, *P* = 0.017, *N* = 32; monkey F = −0.60, *P* = 0.0015, *N* = 26). This trend naturally arises when subjects use the available evidence in a statistically optimal fashion^37,38^.

**Extended Data Fig. 10:**
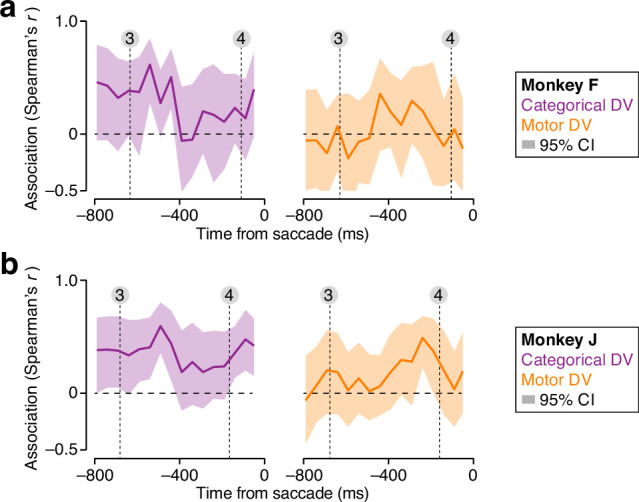
Temporal evolution of the association between a neural measure of expectation and behavioral decision bias. (**a**) We performed the analysis shown in Fig. 6d of the main paper using a sliding 50 ms wide counting window. The full line shows the cross-session correlation between the neural measure of expectation and behavioral decision bias. For both monkeys, the association between neural expectation calculated from the categorical DV and the behaviorally measured bias was substantial around the time of stimulus onset (indicated by the leftmost dotted line), but decreased as the trial progressed. The association between neural expectation calculated from the motor DV and behavioral bias was minimal around the time of stimulus onset, but gradually increased in strength as the trial progressed. Confidence intervals are based on a 10,000 fold bootstrap test. (**b**) Same as (**a**) for monkey J.

To isolate the effects of the monkeys’ prior knowledge on the neural representation, we compared DV trajectories of trials that resulted in the same categorical choice but that were either congruent or incongruent with the prior expectation (Methods). As can be seen from an example recording session, congruent and incongruent categorical DV trajectories could differ substantially (Fig. 6c, top left). This difference, which we term DV bias, was often present before stimulus onset and was more prominent during blocks of low-contrast trials (Fig. 6c, bottom left). This suggests that it may provide a neural measure of the impact of prior expectations on ensuing perceptual decisions. To test this idea, we calculated the DV bias around the time when the categorical DV first begins to reflect stimulus information (that is, 500 ms before saccade initiation; Fig. 6c, bottom left, red arrows). For every recording session, we thus obtained two neural measures of ‘expectation’, one for high-contrast trials, and one for low-contrast trials. For both monkeys, expectation calculated from the categorical DV predicted the behaviorally measured decision bias (Fig. 6d; left, Spearman rank correlation: monkey J = 0.59, *P* < 0.001; monkey F = 0.61, *P* = 0.0011). For the motor DV, this was not the case (Fig. 6d; right, monkey J = 0.025, *P* = 0.89; monkey F = −0.067, *P* = 0.74). Calculating neural expectation from slightly earlier or later moments in time yielded similar results (Extended Data Fig. 10). These results further corroborate the hypothesis that deliberation occurred in an abstract stimulus representation space. They also imply that, during perceptual deliberation, PFC activity is shaped not only by input from visual cortex, but also by signals representing prior knowledge retrieved from memory.

Finally, we asked whether stimulus contrast impacted the DV trajectories. Lowering contrast delayed the onset of the categorical DV by 15 ms (*P* = 0.12, two-sided Wilcoxon signed-rank test; Supplementary Fig. 4). Although not statistically significant, such delay may be inherited from sensory cortex, as V1 neurons have longer response latencies at low stimulus contrast^39^. We did not find any other significant effects of contrast (Supplementary Fig. 4).

## Discussion

In this study, we investigated neural population activity in PFC during flexible orientation discrimination. We sought to probe the nature of the decision process that underlies the flexible relationship between perception and action demanded by many of the real-world problems we face. We suggest that behavioral reports arise from a decision process in which evaluating the sensory environment and planning to act on that interpretation are supported by the same populations of neurons, but unfold in separate representational spaces and different moments in time (Fig. 5a). This interpretation is supported by three distinct sets of observations. First, during sensory stimulation, an initial population representation of the upcoming categorical choice precedes an orthogonal representation of the motor action used to communicate that choice (Figs. 2 and 3). Second, neural activity patterns predictive of the upcoming categorical choice reflect a graded representation of evidence, temporal diffusion and the presence of a bound, while activity patterns predictive of the upcoming motor response do not (Fig. 4). Third, prior stimulus expectations shape the formation of the categorical choice but not the formation of the action plan (Fig. 6).

Our investigation directly compares population representations of perceptual deliberation and motor planning as they unfold together in orthogonal spaces^12^. Previous attempts to determine whether action-planning circuits in the macaque brain also support abstract perceptual deliberation were inconclusive for a variety of reasons. Some studies used a temporal match-to-sample task^7,40–43^. In these tasks, the DV consists of a comparison of two stimulus representations. As a consequence, such tasks allow for the identification of abstract perceptual representations^7,40–43^, but not for the identification of neural deliberation signals. Some other studies used a task design similar to ours, but found that animals appeared to adopt an intentional strategy and that neural activity did not reflect categorical choice formation^44,45^. Compared with the present work, these studies involved judgment of different stimulus features (checkerboard color^44^ and random dot motion^45^), recordings from different brain areas (premotor cortex^44^ and area LIP^45^), a different order of task events (choice targets appeared at an unpredictable location after the offset of the stimulus^45^), and probably a different training history (our monkeys first learned to flexibly report perceptual judgments and only then became experts in orientation discrimination). These multidimensional differences prohibit an unambiguous interpretation of the cross-study differences in choice-related neural activity. Finally, in most previous studies, neural signals were recorded from one unit at a time and could thus not reveal the structure of population activity^11,19,42^. We believe that our findings may offer useful insight into human perceptual decision-making. A recent study of magnetoencephalography signals in the human brain during flexible motion discrimination found strong evidence for abstract perceptual choice signals^46^.

We targeted the prearcuate gyrus because it receives inputs from visual cortex and projects to premotor and motor cortex. Neurons in this area are implicated in both sensory and motor function^14^, making it a likely candidate for sensorimotor transformations like those required for our task^2,9,15^. To interpret neural activity, we compared the structure of choice-related signals with a theoretical decision process in which bounded accumulation in an abstract perceptual dimension is followed by rotation onto an orthogonal motor preparation dimension. In our view, this specific theoretical decision process offers a useful point of reference because it describes a principled solution for the general problem of choosing how to act in a world that is inherently uncertain^12,28,38,47^. That said, our analysis raises many new questions. Can this theoretical ideal quantitatively describe spiking activity in PFC? How exactly is the mapping rule steering the hypothesized rotation? And under which training conditions does a neural network implement this decision process? Answering these questions will require careful comparison of our data with predictions of artificial neural networks capable to perform complex decision-making tasks—an approach that has been particularly fruitful in the study of PFC in recent years^15,18,48,49^.

A strong version of the abstract hypothesis holds that deliberation exclusively unfolds in the categorical dimension and that motor planning does not begin until deliberation is complete. At face value, the temporal overlap in the rise of the average categorical and motor DV trajectories is inconsistent with a pure sequential process (Fig. 2e). However, this inconsistency is difficult to judge. The hypothesis pertains to the single trial level. Due to cross-trial differences in the onset and duration of the deliberation process, the average trajectories can have properties not present at the single trial level^50,51^, including an illusory temporal overlap between both dimensions. Fully evaluating whether the timecourse of the DV trajectories is well described by a sequential process will thus require a detailed investigation at the single trial level.

Decision-related activity has been found in many different brain areas^47^. It has been challenging to ascribe a unique role to each of these areas. This requires experimental paradigms that are simple enough to invite well-controlled reliable behavior, but complex enough to engage higher cognitive mechanisms. Our paradigm revealed dissociable signatures of stimulus strength, perceptual uncertainty, prior knowledge and action plans within a single area. Rich perceptual decision-making tasks in which perceptual and motor components of the decision process are orthogonalized therefore hold promise to disambiguate the functional roles of brain areas within the decision-making network, and, more generally, to characterize the cascade of neural operations that collectively transform sensory inputs into perceptual interpretations and context-appropriate action plans.

## Methods

### Subjects

Our experiments were performed on two adult male macaque monkeys (*Macaca mulatta*, aged 8–9 years old over the course of the experiments). The animals were trained to perform a memory-guided saccade task and an orientation discrimination task with saccadic eye movements as operant responses. They had not previously participated in research studies. All training, surgery and recording procedures conformed to the National Institute of Health Guide for the Care and Use of Laboratory Animals and were approved by The University of Texas at Austin Institutional Animal Care and Use Committee. Under general anesthesia, both animals were implanted with three custom-designed titanium head posts and a titanium recording chamber^52^.

### Apparatus

The subjects were seated in a custom-designed primate chair in front of a CRT monitor (Sony Trinitron, model GDM-FW900), with their heads restrained using three surgical implants. Stimuli were shown on the CRT monitor, which was positioned approximately 64 cm away from the monkeys’ heads. Eye position was tracked continuously with an infrared eye tracking system at 1 kHz (Eyelink 1000, SR Research). Stimuli were generated using the Psychophysics Toolbox^53^ in MATLAB (MathWorks). Neural activity was recorded using the Plexon OmniPlex System (Plexon). Precise temporal registration of task events and neural activity was obtained through a Datapixx system (Vpixx). All of these systems were integrated using the PLDAPS software package^54^.

### Memory-guided saccade task

We used a variation of the classical memory-guided saccade task^55^ to identify recording sites where neurons exhibited neural activity indicative of an upcoming eye movement. Each trial began when the subject fixated a small white square at the center of the screen. After 100 ms, a small response target briefly appeared in 1 of 24 possible locations (3 radii × 8 directions). The subject needed to keep this location in memory while maintaining fixation for 500 ms. After this delay period, the fixation mark disappeared and the subject needed to make a saccade to the remembered location. Correct choices were followed by a juice reward. Each location was presented several times per recording session.

### Estimating response field locations

During the memory-guided saccade task, extracellular recordings were made with dura-penetrating glass-coated tungsten microelectrodes (Alpha Omega), advanced mechanically into the brain. We made recordings from several sites in the prearcuate gyrus. After data collection was completed, we studied spiking activity in a 100-ms window preceding saccade initiation. We compared the strength of the response preceding an eye movement to the neuron’s apparent preferred spatial location with the responses preceding eye movements to all other locations. We deemed a neuron to have a well-defined motor response field if this difference fell outside the expected difference distribution predicted by a null-model that assumes Poisson spiking statistics. Following identification of a suitable recording site (Extended Data Fig. 1), we conducted several additional orientation discrimination training sessions with one choice target placed within the estimated response field location and one on the opposite site of the fixation mark. Once psychophysical performance reached a high level, physiological data collection began.

### Orientation discrimination task

The orientation discrimination task is a variant of classical visual categorization tasks in which the subject uses a saccadic eye movement as operant response^56–58^. We used a flexible version of this task in which the stimulus-response mapping rule varied from trial to trial^10,11,19,44,45,59,60^. Each trial began when the subject fixated a small white square at the center of the screen (0.6 degrees in diameter). Upon fixation, the square was replaced by either a triangular or a circular fixation mark, indicating the latent prior context of the trial. The experiment involved two distinct prior contexts, associated with differently skewed distributions of stimulus orientation (inset, Fig. 6a). Blocks of both priors alternated randomly (80 trials per block). At 500 ms + 0–65 ms after the onset of the fixation mark, two choice targets appeared, one on each side of the fixation mark. One choice target was placed within the presumed motor response field, the other on the opposite side of the fixation mark. The choice targets were white lines (2.5 degrees × 0.5 degrees), rotated −22.5 degrees and 22.5 degrees from vertical. At 250 ms + 0–65 ms later, a circularly vignetted drifting grating appeared in the near periphery (eccentricity: 1.12 degrees). The grating measured 2.7 degrees in diameter, had a spatial frequency of one cycle per degree and a temporal frequency of one cycle s^−1^. The stimulus remained on for 500 ms + 0–65 ms. Subjects judged the orientation of the stimulus relative to vertical. The stimulus then disappeared along with the fixation mark and subjects reported their decision with a saccadic eye movement to the appropriately oriented choice target. Trials in which the monkey did not saccade to either of the choice targets within 2 s were aborted. Auditory feedback about the accuracy of the monkey’s response was given at the end of each trial. Correct choices were followed by a liquid reward delivered via a solenoid-operated reward system (New Era). Stimuli were seven drifting gratings evenly spaced over a small range of orientation, tailored to each monkey’s orientation sensitivity (monkey F, −3.75 degrees to 3.75 degrees, monkey J: −3.3 degrees to 3.3 degrees). Vertically oriented stimuli received random feedback. Stimuli were presented at either high or low contrast (Michelson contrast, 100% or 4%). Blocks of high- and low-contrast stimuli alternated randomly (trials per block, monkey F = 100; monkey J = 80). We conducted 13 successful recordings from monkey F and 16 from monkey J (average number of trials per session, monkey J = 3,171; monkey F = 1,593).

### Behavioral analysis

We measured observers’ behavioral capability to discriminate stimulus orientation by fitting the relationship between stimulus orientation and probability of a ‘clockwise’ choice with a psychometric function consisting of a lapse rate and a cumulative Gaussian function. Model parameters were optimized by maximizing the likelihood over the observed data, assuming responses arise from a Bernoulli process. Each recording session was analyzed independently. For the analysis documented in Fig. 1d, we fit one psychometric function per mapping rule and contrast level. We defined orientation sensitivity as the inverse of the standard deviation (s.d.) of the cumulative Gaussian. We used a variant of this model to measure observers’ prior-induced behavioral decision bias. For this analysis, we fit one psychometric function per stimulus prior and contrast level (Fig. 6a). Both prior conditions shared the same sensitivity parameter, resulting in two psychometric functions with identical slope. We defined decision bias as the difference between the means of both cumulative Gaussians (that is, the magnitude of the horizontal displacement of both psychometric functions). Error bars of model-based statistics are based on a 100-fold nonparametric bootstrap of the behavioral data.

### Electrophysiological recordings

During the orientation discrimination task, we recorded extracellular spiking activity from populations of PFC neurons through a chronically implanted recording chamber. Every recording session, we used a microdrive (Thomas recording) to mechanically advance a linear electrode array (Plexon S-probe; 32 contacts) into the brain at an angle approximately perpendicular to the cortical surface (Extended Data Fig. 1). We targeted recording sites that had exhibited well-defined motor response fields in a previously conducted memory-guided saccade task. We positioned the linear arrays so that they roughly spanned the cortical sheet and removed them after each recording session. Continuous neural data were acquired and saved to disk from each channel (sampling rate 30 kHz, Plexon OmniPlex System). To extract responses of individual units, we performed offline spike sorting. We first automatically spike-sorted the data with Kilosort^61^, followed by manual merging and splitting as needed. Given that the electrode’s position could not be optimized for all contact sites, most of our units probably consist of multineuron clusters. All units whose mean firing rate during the task exceeded 3 ips were included in the analysis.

### Analysis of single-unit responses

We measured the temporal evolution of each unit’s response by expressing spike times relative to the trial-specific moment of saccade initiation and counting spikes within nonoverlapping 50-ms windows. Figure 2a shows example response traces for four units, averaged across different subsets of trials. We computed neuronal selectivity for the upcoming choice behavior by calculating the difference between the choice-conditioned response averages, normalized by the response standard deviation^62^. The sign of this signal-to-noise (SNR) metric depends on the unit’s preferred choice option. To facilitate comparison across the categorical and motor dimension, we signed each unit’s SNR-trace such that the maximal value was positive (see examples in Fig. 2a; all traces are shown in Fig. 2b).

### Estimating the time-varying DV

For each trial, we obtained moment-to-moment measurements of the DV by projecting 50-ms bins of population activity onto a linear decoder optimized to distinguish the activity patterns associated with both choice options (‘left’ versus ‘right’ choices for the motor DV, and ‘clockwise’ versus ‘counterclockwise’ choices for the categorical DV, respectively). Specifically, we first individually *z*-scored each unit’s spike counts within every time bin. We then used these *z*-scored responses to estimate the set of linear weights, *w* = (*w*_1_,…, *w*_*n*_), that best separate the choice-conditioned *z*-scored response patterns, assuming a multivariate Gaussian response distribution:

1 $$w=\frac{s}{\Sigma }$$

where *s* is the mean difference of the choice-conditioned *z*-scored responses and $\Sigma$ is the covariance matrix of the *z*-scored responses. To quantify overall decoder performance, we used the same procedure to estimate pooling weights but used a single 750-ms counting window (Extended Data Fig. 3). The decoder weights are calculated from observed trials. To avoid double-dipping, we excluded the trial under consideration from the calculation and solely used all other trials to estimate the weights. This way, we obtained ‘cross-validated’ DV estimates for each time bin:

2 $${{\mathrm{DV}}}_j=\sum {w}_{ij}{Z}_{ij}$$

where ${w}_{ij}$ and ${Z}_{ij}$ are the weight and *z*-scored response of unit *i* on trial *j* for a given time bin. The curves in Extended Data Fig. 4 show example single trial DV trajectories. The symbols in Fig. 2d show DV trajectories from three example recording sessions, averaged across all choice-conditioned trials. The symbols in Fig. 3a show DV trajectories from the same example recording sessions for the zero-signal stimulus. The lines in Fig. 2e and Fig. 3b show unsigned DV trajectories, obtained by inverting the trajectories associated with ‘counterclockwise’ and ‘left’ choices and grouping these with the ‘clockwise’ and ‘right’ trajectories, respectively. The lines in Fig. 4b show unsigned DV trajectories, split by stimulus strength and choice accuracy, and averaged across all recording sessions of both animals. The lines in the top panel of Fig. 6c show unsigned DV trajectories of an example recording session averaged across choice ‘congruent’ and ‘incongruent’ trials, respectively.

### Comparison of DVs

We compared the orientation of the categorical and motor dimensions by computing the average vector angle between the associated projection planes. For each recording session, we calculated the vector angle $\theta$:

3 $$\theta ={\cos }^{-1}\left(\frac{{\sum }_{i}{w}_{{{i{\mathrm{c}}}}}{w}_{{{i{\mathrm{m}}}}}}{\sqrt{{\sum }_{i}{w}_{{{i{\mathrm{c}}}}}^{2}}\sqrt{{\sum }_{i}{w}_{{{i{\mathrm{m}}}}}^{2}}}\right)$$

where *w*_*i*c_ and *w*_*i*m_ are the weights of unit *i* used to compute the categorical and motor DV for a given time bin. Figure 5b shows the average vector angle (circular mean) across recording sessions. We used the same approach to measure the temporal stability of each projection plane (Extended Data Fig. 9).

To quantify the similarity of the categorical and motor DV, we computed the normalized cross-correlation function for every trial within a given recording session. We then averaged these functions across trials, split by mapping rule. For some recording sessions, this procedure resulted in two average cross-correlation functions that exhibited similar idiosyncratic features (for example, ripples, or an oddly placed peak). These features are probably meaningless and arise simply because the DVs are themselves autocorrelated^63^. To focus our analysis on systematic differences across mapping rules, we therefore subtracted each recording sessions’ global average cross-correlation function from each mapping rule’s specific average (Fig. 5c, lines). This correction procedure was effective, but may itself give rise to spurious mapping rule differences. We therefore performed the same analysis on a shuffled version of the data to quantify chance expectation (Fig. 5c, bands).

### Analysis of DV variance

We measured the effect of stimulus strength on the categorical and motor DV by computing the fraction of variance explained by this independent variable. As is standard in analysis of variance, one can partition the total sum of squares (*S*_total_) into components arising from variations in choice outcome (*S*_choice_), stimulus strength (*S*_stim_) and residual variance (*S*_res_):

4 $${{S}}_{{\rm{total}}}={{S}}_{{\rm{choice}}}+{{S}}_{{\rm{stim}}}+{{S}}_{{\rm{res}}}$$

5 $$\begin{array}{rcl}\sum _{{k}}({{\mathrm{DV}}}_{{k}}-\overline{{\mathrm{DV}}})^{2}&=&\sum _{{k}}({\overline{{\mathrm{DV}}}}_{{k}}-\overline{{\mathrm{DV}}})^{2}\\&&+\sum _{{k}}({\overline{{\mathrm{DV}}}}_{\mathrm{s}},_k-\,{\overline{{\mathrm{DV}}}}_{{k}})^{2}\\&&+{\sum }_{{k}}({{\mathrm{DV}}}_{{k}}-{\overline{{\mathrm{DV}}}}_{{{\mathrm{s}},k}})^{2}\end{array}$$

where DV_*k*_ is the DV value on the *k*th trial, ${\overline{{\mathrm{DV}}}}_{{{\mathrm{s}},k}}$ the DV averaged over those trials in which the presented stimulus and choice outcome were the same as those of the *k*th trial, ${\overline{\mathrm{{DV}}}_{k}}$ the DV averaged over those trials in which the choice outcome was the same as that of the *k*th trial, and $\overline{{\mathrm{DV}}}$ the DV averaged over all trials. The temporal evolution of the cross-session averaged ratio between the component due to stimulus strength and the total is plotted in Extended Data Fig. 8, and that of the ratio between the residual and the total is plotted in Fig. 4d.

### Descriptive models of computational hypotheses

We compared the observed DV trajectories with the theoretical expectations of three computational models of decision-making. We expressed the models’ predictions using a set of equations that describe the average evolution of the choice-conditioned DV. Under the intentional model, the categorical DV has no systematic structure, whereas the motor DV evolves according to a cumulative Gaussian function. This model has four free parameters per choice-conditioned trajectory: one captures an initial offset in the motor DV, one specifies the dynamic range of the DV trajectory, one controls the speed of the rise and one the timepoint at which half of the rise is completed. Under the abstract model, an initial rise in the categorical DV is followed by a subsequent rise of the motor DV. Following completion of the deliberation process, the categorical DV may decay in strength (exponential decay). We used nine free parameters to describe this pattern. Five of these specify the evolution of the categorical DV, and four that of the motor DV. For both DVs, we used cumulative Gaussians in the same way as we did for the intentional model. For the categorical DV, we additionally used a parameter that controls the amount of decay that follows the peak of the categorical DV (defined as the time at which the cumulative Gaussian reached the 99.38th percentile). We imposed boundaries on the model’s parameters that ensured that the motor DV could not begin to rise before the categorical DV. Specifically, the bounds ensured that the motor DV could not reach its half-maximum value before at least 250 ms had passed since the categorical DV reached its half-maximum value. Under the mixture model, the speed of the rise and the timepoint at which half the rise is completed is identical across both the categorical dimension. We used seven free parameters to describe this pattern. We fit all three descriptive models by minimizing the sum of the squared error of the choice-conditioned trajectory under consideration. Example fits of the abstract model are shown in Fig. 2d and Fig. 3a, and example fits of the intentional model are shown in Extended Data Fig. 7. To compare the models’ goodness of fit, we computed an estimator of prediction error based on information theory (Akaike information criterion (AIC)). Specifically, assuming that the residuals under each model are distributed according to independent identical normal distributions:

6 $${\rm{AIC}}=2k+{\it{n}}{\rm{ln}}({\hat{\sigma }}^{2})-2{\mathrm{C}}$$

where *k* is the number of free parameters, *n* the number of data points, C a constant that only depends on the data, and ${\hat{\sigma }}^{2}$ the maximum likelihood estimate for the variance of a model’s residuals distribution given by the residual sum of squares divided by the degree of freedom. Because only differences in AIC are meaningful, the constant C can be ignored when comparing models, yielding a statistic known as $\Delta$AIC. A comparison of this statistic for the abstract and intentional model is plotted in Fig. 2d and Fig. 3a.

### Estimating onset and peak time of DV trajectories

We conducted an analysis in which we compared the estimated onset time of both DVs (Figs. 2f and 3c). We obtained estimates of onset time by fitting an unconstrained version of the abstract model to the data. This model used the same set of equations as the abstract model, but we imposed no boundaries on the model’s parameters that would enforce a temporal order on the DV trajectories. The average fit of this model to the data is shown in Extended Data Fig. 6a. For each DV trajectory, we defined onset time as the time at which the cumulative Gaussian reached the fifth percentile. Because the dynamic range of the categorical and motor DV can differ, this quantification defines onset time in relative terms. We complemented this analysis with a quantification that defines onset time in absolute terms, independent of the specific trajectory’s dynamic range. These estimates are shown in Extended Data Fig. 7. We also conducted an analysis in which we compared the estimated peak time of the categorical and motor DV for different groups of trials (Fig. 4f). We obtained estimates of peak time by fitting the same unconstrained version of the model to each trajectory shown in Fig. 4b. The fits are shown in Extended Data Fig. 8b. Under this model, peak time is defined as the time at which the cumulative Gaussian reaches the 99.38th percentile (at this time, the decay begins). We obtained estimates of the standard error by repeating this analysis on 1,000 matching synthetic datasets, each created by sampling the observed trials with replacement. We then performed the entire analysis sequence on these bootstrapped trials. The error bars in Fig. 4f show the estimate for the observed data ±1 s.d. of the peak time estimates of the synthetic datasets.

### Estimating DV bias

We obtained estimates of DV bias by first calculating the average observed unsigned DV trajectory for congruent and incongruent trials per level of stimulus strength (that is, rotation magnitude), then taking the difference of these averages per level, and finally averaging across these differences. This estimation procedure ensures that stimulus strength as such does not impact the bias estimate (the fraction of congruent and incongruent choices differs across stimulus strengths).

### Statistics and reproducibility

No statistical methods were used to predetermine sample sizes but our sample sizes are similar to those reported in previous publications^44,45,51^. Data collection and analysis were not performed blind to the conditions of the experiments.

### Reporting summary

Further information on research design is available in the Nature Portfolio Reporting Summary linked to this article.

## Online content

Any methods, additional references, Nature Portfolio reporting summaries, source data, extended data, supplementary information, acknowledgements, peer review information; details of author contributions and competing interests; and statements of data and code availability are available at 10.1038/s41593-024-01635-1.

### Supplementary information

Supplementary Information Supplementary Figs. 1–4.

Reporting Summary

## Data Availability

The data that support the findings of this study are available from the corresponding author upon request. The authors are conducting further analyses of the data. The data will be made publicly available once this has been completed.

## Code availability

The code used to generate the results presented in this paper is available from the corresponding author upon request.
