# Supplementary material for: Abstract deliberation by visuomotor neurons in prefrontal cortex
Source: Nat Neurosci. 2024 Apr 29;27(6):1167–75. doi: 10.1038/s41593-024-01635-1 (PMC11156582; doi:10.1038/s41593-024-01635-1)
Supplement: Supplementary file 1 — Supplementary Figs. 1–4. [file 41593_2024_1635_MOESM1_ESM.pdf]

nature neuroscience

Article https://doi.org/10.1038/s41593-024-01635-1

# Abstract deliberation by visuomotor neurons in prefrontal cortex

In the format provided by the authors and unedited

## Supplementary Figures

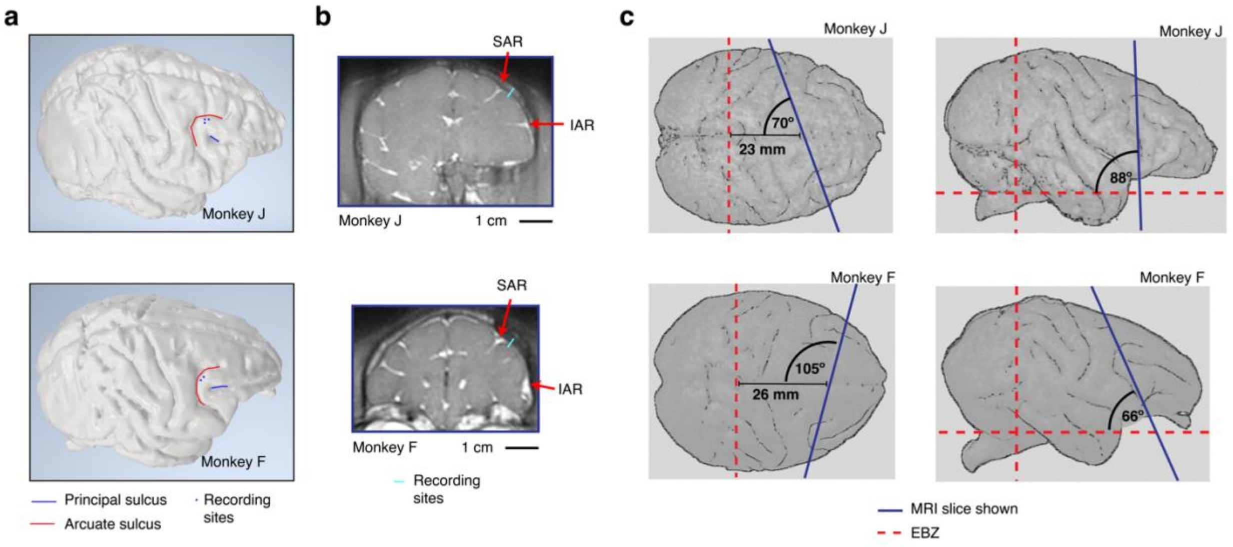


**Supplemental Figure 1.** Location of prearcuate gyrus recordings for monkey J and F (top vs bottom). (**a**) 3D reconstruction of the brain based on a structural MRI scan obtained before chamber and post implants. The location of the recording sites are marked by blue dots. (**b**) Structural MRI scan illustrating the approximate recording site. The arrows indicate the superior arcuate sulcus (SAR) and inferior arcuate sulcus (IAR). (**c**) Top and side view (left vs right) of the MRI slice shown in panel **b**.

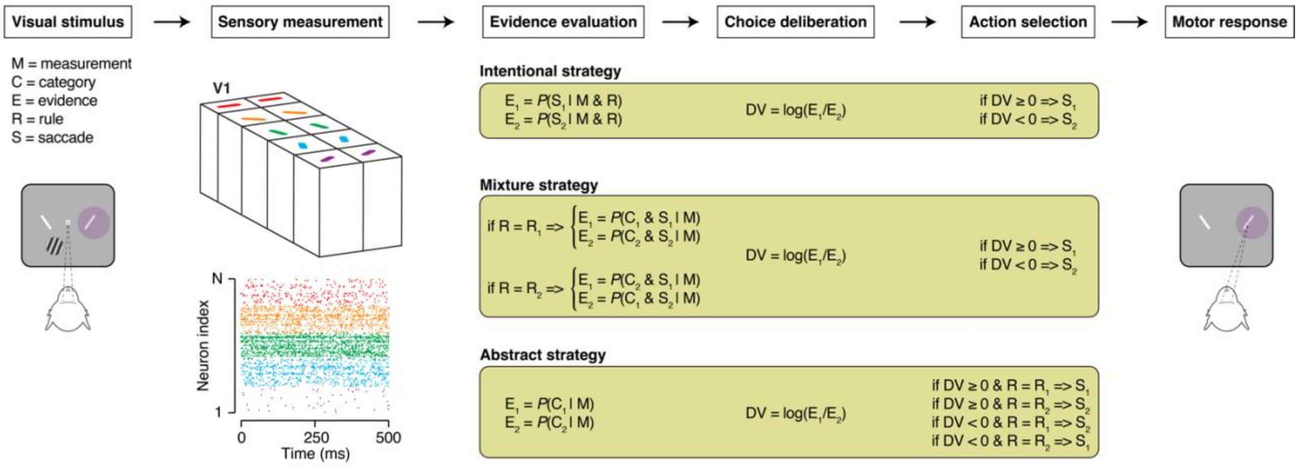


**Supplemental Figure 2.** Further comparison of candidate computational strategies. The sensorimotor transformation underlying flexible visual categorization in our task can be broken down into a sequence of conceptually distinct operations (top, boxes). Common to all three candidate strategies is that information about stimulus orientation must be obtained from a sensory measurement (left part of diagram) and that the decision must be communicated with a saccadic eye movement (right part of diagram). The sensory measurement is likely provided by the population activity of a set of visual neurons whose responses selectively depend on stimulus orientation (e.g., by the collective output of a cortical hypercolumn in primary visual cortex). Under an intentional strategy, this activity is evaluated by converting it into evidence in favor of each possible motor response (E1 and E2, which ideally reflect the likelihood of each response option being correct). This transformation requires taking into account the trial-specific mapping rule. Under an abstract strategy, the sensory activity is evaluated by converting it into evidence in favor of each possible categorical response. This transformation does not require knowledge of the mapping rule. Under a mixture strategy, sensory activity is transformed into evidence favoring one of two possible combinations of categorical choice and associated saccade option. The mapping rule determines the trial-specific pair of combinations. Under all three strategies, choice deliberation involves comparing the evidence in favor of each response option. The logarithm of the likelihood ratio provides a principled metric for this operation. Under the intentional and mixture strategy, the deliberation process directly results in a motor plan. Under the abstract strategy, following deliberation, the mapping rule must be consulted to form the appropriate motor plan.

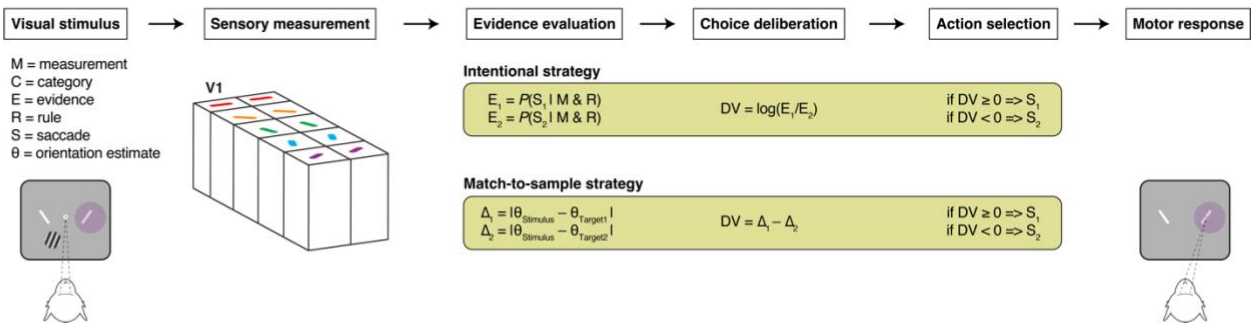


**Supplemental Figure 3.** Further comparison of candidate computational strategies. In principle, the subject could solve the task using a spatial match-to-sample strategy. Under this strategy, the perceived stimulus orientation is compared with the orientation of both choice targets, and the most similarly oriented choice target is selected. This strategy is a task-specific variant of an intentional strategy in the sense that the deliberation concerns the question of whether one possible saccade response is favored over the other possible saccade response. Like the intentional hypothesis discussed in the paper, this strategy predicts a data pattern incompatible with our analysis. Specifically, the same stimulus orientation should give rise to oppositely signed DV values under both mapping rules. As documented in the paper, we only see evidence for such a pattern late in the trial, and this pattern does not exhibit neural signatures of deliberation.

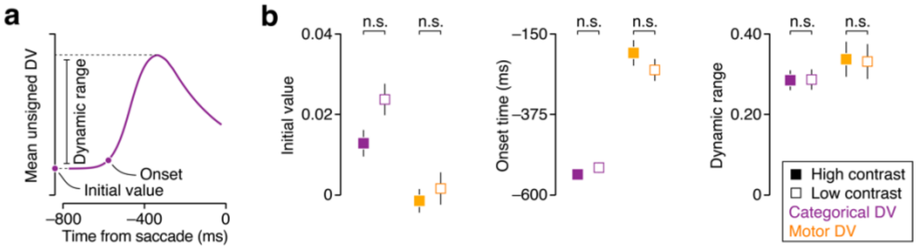


**Supplemental Figure 4.** Effect of stimulus contrast on the unsigned categorical and motor DV. (**a**) For each recording session, we separately computed the four trajectories associated with high and low contrast trials. For each trajectory, we estimated the DV's initial value, relative onset time, and dynamic range. (**b**) Comparison of these statistics for high and low contrast trials (filled vs open symbols). Estimates were pooled across all recording sessions. Categorical and motor DV are shown in purple and orange. Symbols illustrate the mean value computed across all unsigned DV trajectories (N = 116), the error bars indicate +/– 1 standard error of the mean, and the statistical symbols summarize the outcome of a 2-sided Wilcoxon signed rank test. * $P < 0.05$; n.s. not significant. Exact P values, from left to right were 0.06, 0.41, 0.12, 0.31, 0.99, and 0.91.
